# Supplementary material for: Spatial heterogeneity of internal migration in China: The role of economic, social and environmental characteristics
Source: PLoS One. 2022 Nov 17;17(11):e0276992. doi: 10.1371/journal.pone.0276992 (PMC9671463; doi:10.1371/journal.pone.0276992)
Supplement: Attachment — Submitted filename: 1 Response to Reviewers.docx [file pone.0276992.s001.docx]

**Spatial heterogeneity of internal migration in China: the role of economic,**

**social and environmental characteristics**

Dear Editor –

We would like to thank all comments provided by the reviewers in relation to our paper submitted to PLOS ONE. We thank you for the opportunity to submit a revised version of the paper.

We try to incorporate the suggestions as best as possible and explain the doubts more clearly. We believe that the current version of the manuscript has improved greatly based thanks to comments and suggestions received. Listed below are detailed comments and answers on the reviewers’ suggestions/comments. We highlight our responses in red.

Best regards,

Authors

**Changes in the original text are in RED.**

**Reviewer 1**

Reviewer comments

The authors researched and discussed a very interesting, important and topical issue.

We appreciate the comments, suggestions, and careful reading. We try to incorporate the suggestions as best as possible and explain the doubts more clearly. We believe that the current version of the manuscript has improved greatly based on your comments and suggestions.

1. Therefore, the question should be asked whether the authors have studied what are the main factors on internal migration of Eastern and west, for example, for work or family life.

**A:** Thank you for your comment and suggestion. The main purpose of this article is to analyze the characteristics of internal migration at the county-level, which potentially influenced environmental and socioeconomic factors. The GWR methodology explored the spatial variation of internal migration and the related drivers by establishing local regression equations at each point in the study area.

In the conclusion section, we discussed the effect of education level on internal migration, dividing China into the eastern coastal region and the western inland region. In the eastern provinces, areas with higher education level attracted more people to immigration, while in the western provinces, areas with high education level faced out-migration. This indirectly indicates that the out-migration from the western region, especially the out-migration of highly educated people, is mainly due to work reasons.

However, in the analysis of the effects of GDP, population density and labor force on internal migration, we found that areas with higher economic size and labor force level in southern provinces of China were more attractive for immigration. As for population density, which reflects population concentration level, the greatest effect on immigration occurs in the Yangtze River Basin region. In other words, our study was not limited to exploring internal migration in the East and West.

2. Section Methods lacks a quantitative analysis of the use of different modes to better understand temporal and spatial changes of the Chinese migration. Also not stated is what criterion indicates this: OLS and GWR, etc.

**A:** Thank you for your suggestion. We opted to maintain the format in this way, since an analysis of the data for each of the methodologies used in the paper is carried out in the results section. However, for clarification purposes, we have made an adjustment to the text (results section).

OLS method is used to explore the relationship between internal migration and potential influencing factors at the global scale, and the residual sum of squares was minimized for estimation. GWR method is used to explore the spatial heterogeneity of potential influencing factors on internal migration.

… The Gauss function method was chosen for spatial weight, because the Gauss function method could choose a continuous monotonically decreasing function to represent the relationship between weight and distance, which overcomes the shortcoming of the inverse distance method assuming stronger correlation for close features. Bandwidth value was based on AICc value to automatically find the optimal actual distance or adjacent elements.

3. In the POLICY RECOMMENDATIONS, it is possible to prioritize in the two perspectives discussed - macro and micro - e.g. which activities are most important, e.g. population migration on a large scale in Urban & Rural, eastern & west.

**A:** Thank you for your suggestion. We have extended the discussion in this part of the manuscript.

The main characteristics of population migration in China are from rural to urban areas; and from inland to coastal areas. However, the excessive concentration of population on the coast is not conducive to the balanced development of population in space. This paper not only analysed the potential factors, but also the different degrees and levels to which these factors affecting internal migration in space.

With the migration, especially the labour force, to the densely populated urban areas with economies of scale, the population pressure in the vast rural areas will be alleviated, as young adults seek new development in urban areas, the rural population will become older. On the macro level, the government needs to guide the migration of rural population to cities in a planned manner. Specifically, for mega-cities represented by provincial capitals and above, which have spillover effects on immigration to surrounding areas, the government needs to actively promote the construction of independent comprehensive node cities in suburban areas, accelerate the integrated development of urban clusters, and reasonably control the population density of central cities. For the central cities, represented by prefecture-level cities with the tendency of absorption of immigrants from the surrounding areas, it is necessary to break the urban-rural dualism, realize urban-rural integration, promote the new urbanization and rural revitalization in concert, and promote the integrated development of urban and rural areas.

The economic development of the western region lags behind that of the eastern coastal region. The well-educated people in the western region may be motivated to migrate to the eastern region for higher salaries. In the end, the educational gap between the east and the west will continue to widen. On the macro level, the government needs to rationally guide the redistribution of population in eastern, central and western China. On the one hand, the eastern coastal regions have the advantage of talent concentration, especially the Yangtze River Delta, Pearl River Delta and Beijing-Tianjin regions. The eastern coastal regions can adjust the relevant settlement policies (Huko policies) to create high-quality talent centers and innovation highlands. The central and western inland regions should also do their best to "retain local talents". In view of the potential of immigration in densely populated areas of central and western China, several urban clusters of central and western China should be developed to absorb the local migration in central and western China. At the same time, the governments can consider the placement of densely populated industries with scale advantages in the central region and develop industries with local characteristics.

Large-scale urban-rural population migration in China can release the pressure of human activities on natural ecosystems in rural areas and promote the increase of forest cover and biomass. The "peasant-to-citizen" approach reduces the dependence of rural populations on arable land, and this study shows that populations, especially in the east, tend to migrate to areas with high forest cover. Therefore, in terms of policy, a large amount of low-yielding sloping arable land in rural areas can be considered for conversion into forestland. By optimizing the spatial development pattern of the country, green mountains and clear water are equal to mountains of gold and silver.

**Reviewer 2**

Reviewer comments

This study calculated the internal migration, analyzed its spatial characteristics, and discussed the influencing factors of economy, demography, environment, location and transportation, land use, etc. This study is interesting and novel, and has great significance for the population management, industrial layout and population management for the policy-makers in China. I recommend publishing after minor revisions.

We appreciate the comments, suggestions, and careful reading. We try to incorporate the suggestions as best as possible and explain the doubts more clearly. We believe that the current version of the manuscript has improved greatly based on your comments and suggestions.

1. The title of Figures should put below the Figures.

**A:** Thank you for your suggestion. We adjust the text.

2. In the introduction part, the shortcomings of previous studies and the meaning and this paper should be further summary to highlight the novelty.

**A:** Thank you for your comment and suggestion. We have added this information.

The following issues existed in previous research on internal migration. First, the analysis of potential influencing factors of internal migration often suffered from the contradiction between theoretical construction and practical application. Theoretical construction is expected to be comprehensive, while practical application needs to consider the constraints of data accessibility and data covariance. Second, the global regression results between internal migration and related potential factors, such as population density or education level, may have completely opposite findings on their relationship in the analyzed cases in different countries and regions, and thus their spatial heterogeneity must be investigated. Third, some of the studies tend to focus the impact of socioeconomic factors on internal migration and tend to discard the exploration of natural environmental factors, and vice versa. Therefore, the purpose of this paper is to explore the spatial heterogeneity of internal migration in China and to understand the impact of social, economic, and environmental factors on one of the demographic processes.

3. Why the author just use the data of 2000-2010. The population migration may changed greatly after 2010. I encourage the author to explain this point.

**A:** Thank you for your comment and suggestion. We have added an explanation in the limitations section.

Regarding internal migration data, in fact, there are changes after 2010. However, the authoritative source of data on Chinese migration at the counties level is mainly the decennial Chinese census. In other words, the current seventh census data (2020) are still not available. In addition, the reliability and accessibility of county-level migration data from other sources and for other years is highly problematic. Nonetheless, the overall trend of migration from rural to urban areas and from inland to coastal areas in China has not changed fundamentally. Therefore, the relevant conclusions of this paper are still valid.

4. I encourage the author to explain the applicability of the methods used in this study.

**A:** Thank you for your suggestion This study is based on the framework of "theory-modeling-validation-discussion". The theoretical framework is developed by analyzing driving factors of internal migration, selecting appropriate factors from social, economic and environmental aspects and conducting exploratory analyses to form a combination of factors that satisfy the minimum multicollinearity covering the five categories. The spatial heterogeneity of internal migration in China is analyzed at both the global and local levels. The findings and the research methodology are applicable in the context of the general trend of rural-urban and inland-coastal migration in China.

5. The first sentence of the conclusion part should put in introduction part. And the conclusion part should be simplified to highlight the main findings.

**A:** Thank you for your comment and suggestion. The first sentence of the conclusion part appears in the introduction part. Therefore, this sentence has been deleted. Furthermore, we have simplified the conclusion to highlight only the main findings.

6. The cited reference in conclusion part should be deleted. I encourage the author to add a discussion part to compare the results with other researches, and some part of conclusion and the cited references could be changed to discussion part. Moreover, the reason for the different influence of factors in different regions should be further discussed and explained.

**A:** Thank you for your comment and suggestion. We have added a discussion section and made all the suggested changes.

The main characteristics of population migration in China are from rural to urban areas; and inland to coastal areas. Migration had a bigger impact on population change in 2010 than in 2000. This conclusion follows the findings of Bell et al. (2015), where migration played a decisive role in the redistribution of the Chinese population.

Economic agglomeration, population agglomeration and age-appropriate labor agglomeration are often complementary and tend to create scale effects, i.e., population prefers to move to places with high economic and population concentrations. There is a vast literature discussing the role of GDP in migration (Aronsson et al. 2001; Gerber 2006; Biagi, Faggian and McCann 2011; Bell et al. 2015). The areas with larger economy scale often mean better infrastructure, more potential jobs and so on, making them the primary choice for people to move in. From the perspective of China's current development, the agglomeration advantage brought by high population density (such as urban areas) is higher than the pressure brought by population crowding, which promote more people to migrate to areas with higher population density. Areas with a demographic dividend advantage are more likely to attract immigrants. The areas with higher level of GDP, population density and labour force proportion means more people to move in, and thus form the Matthew effect in space (the stronger gets stronger and the weaker get weaker). The areas with these three advantages originally (such as cities or urban agglomeration region) will continue to maintain and strengthen these advantages by immigration, while other areas, such as rural areas, have to face the problems of relatively smaller economy, a lower population density and an aging population society.

As for soil nutrient and land use, some studies believe that it is beneficial to the agricultural population growth, but regions with large agricultural production potential may also be experiencing population loss (Zhang 2011). Areas with favorable agricultural conditions (represented by soil nutrients), carrying more agricultural population, have become the main source of out-migration for better job opportunities. Especially the inland areas with relatively backward economic development.

7. The limitation of this study, I think, should be discussed.

**A:** Thank you for your suggestion. We have included the limitations of the study.

As for the limitations of this study, firstly, this study adopted a stepwise regression method to exclude factors that were less correlated with internal migration and had obvious covariance between factors, which may suggest a lacked a mechanism analysis. Secondly, this study focused on the spatial heterogeneity of potential factors influencing internal migration, but the distribution characteristics and the degree of influence of such spatial heterogeneity were yet to be studied in comparison with relevant empirical studies. Finally, this study did not analyse migration resistance factors, such as migration intention.

Regarding internal migration data, in fact, there are changes after 2010. However, the authoritative source of data on Chinese migration at the counties level is mainly the decennial Chinese census. In other words, the current seventh census data (2020) are still not available. In addition, the reliability and accessibility of county-level migration data from other sources and for other years is highly problematic. Nonetheless, the overall trend of migration from rural to urban areas and from inland to coastal areas in China has not changed fundamentally. Therefore, the relevant conclusions of this paper are still valid.
